# Downregulated Recycling Process but Not De Novo Synthesis of Glutathione Limits Antioxidant Capacity of Erythrocytes in Hypoxia

**DOI:** 10.1155/2020/7834252

**Published:** 2020-09-04

**Authors:** Yueming Wang, Nannan Zhao, Yanlei Xiong, Jiashen Zhang, Dongmei Zhao, Yancun Yin, Lele Song, Yipeng Yin, Jing Wang, Xiying Luan, Yanlian Xiong

**Affiliations:** ^1^Department of Anatomy, School of Basic Medicine, Binzhou Medical University, Yantai, China; ^2^Department of Immunology, School of Basic Medicine, Binzhou Medical University, Yantai, China; ^3^Department of Pathology, Xuanwu Hospital, Capital Medical University, Beijing, China

## Abstract

Red blood cells (RBCs) are susceptible to sustained free radical damage during circulation, while the changes of antioxidant capacity and regulatory mechanism of RBCs under different oxygen gradients remain unclear. Here, we investigated the changes of oxidative damage and antioxidant capacity of RBCs in different oxygen gradients and identified the underlying mechanisms using an in vitro model of the hypoxanthine/xanthine oxidase (HX/XO) system. In the present study, we reported that the hypoxic RBCs showed much higher oxidative stress injury and lower antioxidant capacity compared with normoxic RBCs. In addition, we found that the disturbance of the recycling process, but not de novo synthesis of glutathione (GSH), accounted for the significantly decreased antioxidant capacity of hypoxic RBCs compared to normoxic RBCs. We further elucidated the underlying molecular mechanism by which oxidative phosphorylation of Band 3 blocked the hexose monophosphate pathway (HMP) and decreased NADPH production aggravating the dysfunction of GSH synthesis in hypoxic RBCs under oxidative conditions.

## 1. Introduction

Red blood cells (RBCs) are susceptible to oxidative injury because of high concentrations of molecular oxygen, membrane polyunsaturated fatty acids, and heme-bound and free iron atoms [1]. Changes in the oxidative status of RBCs can reduce cell lifetime, oxygen transport, and delivery capacity to peripheral tissues and have been associated with a great number of human diseases [2, 3]. Previous studies have shown that the changes of hemoglobin (Hb) conformation under different oxygen gradients can affect the antioxidant capacity of RBCs [4]. Recent investigation by Rogers et al. demonstrated that hypoxia limits the antioxidant capacity of RBCs by altering glycolytic pathway dominance [5]. However, under oxidative stress conditions, the relationship between oxygen gradients and the antioxidant capacity of RBCs remains to be elucidated.

Recently, studies report that hypoxia exposure induces free radical oxidative damage in various tissues [6, 7]. Under a variety of physiological and pathological conditions, the reactive oxygen species (ROS) produced by metabolism will attack RBCs, resulting in a series of damages such as Hb oxidation, membrane protein crosslinking, and membrane lipid peroxidation, which causes oxygen carrying and deformability dysfunction of RBCs, and finally becomes a potential pathogenic factor of cardiovascular and cerebrovascular diseases [4, 8]. RBCs possess an excellent antioxidant capacity for the timely removal of ROS from cells, plasma, and tissue [1, 9]. The main antioxidant defense system consists of enzymes such as catalase (CAT), superoxide dismutase (SOD), and numerous nonenzymatic antioxidants including glutathione (GSH), ubiquinone, and flavonoids [10, 11]. However, under oxidative stress conditions, the response of antioxidants to the changes of oxygen gradients in RBCs is still unclear.

The activation and synthesis of most antioxidant enzymes need a constant supply of energy which reduce power; these include ATP, NADPH, and NADH, which are mostly products of glucose metabolism in RBCs [12]. Accumulating evidence indicated that glucose metabolism in RBCs is characterized by O_2_-responsive variations in flux through the Embden-Meyerhof pathway (EMP) or the hexose monophosphate pathway (HMP) [13, 14]. There is evidence that the competitive binding between key EMP enzymes and deoxy-Hb to the N-terminal of Band 3 regulates the glucose metabolism through the EMP or HMP pathway of RBCs [4, 15]. However, under oxidative stress conditions, the binding sites of Band 3 to deoxy-Hb or EMP enzymes may also be damaged, which will lead to the dysfunction of the above regulatory effect. Previous studies on RBC metabolism at high altitude have also shown that steady state levels of glycolytic metabolites increase and levels of HMP intermediates decrease upon exposure to high-altitude hypoxia [16–18]. Oxygen-releasing capacity and glucose metabolism fluxes through EMP or HMP are regulated by hypoxia as a function of purinergic and sphingosine 1 phosphate (S1P) signaling [19, 20]. Hypoxia *in vitro* and *in vivo* decreased purine oxidation and enhanced purine salvage reactions in human and mouse RBCs [21]. Furthermore, oxidative and metabolic lesions, exacerbated by storage under hyperoxic conditions, were ameliorated by hypoxic storage [22–24]. Recently, studies have reported that GSH synthesis of RBCs is impacted by hemoglobin oxygen saturation and intracellular pH [25–27]. Hypoxic storage improves energy metabolism and GSH homeostasis of stored RBCs through a process of recycling but not through synthesis [28]. Therefore, there may be some regulatory mechanisms between the oxygen gradients and the antioxidant capacity of RBCs, so that it can regulate the antioxidant capacity to cope with the differences of oxidative stress risks under different oxygen contents.

Based on these observations, we hypothesized that the antioxidant capacity could be associated with the alterations in the oxygen gradients of RBCs. Hypoxanthine is an *in vitro* metabolic marker of RBC injury [21]. To test our hypothesis, an *in vitro* model of the hypoxanthine/xanthine oxidase (HX/XO) system was used to detect the change of oxidative damage and antioxidant capacity of RBCs under both normoxic and hypoxic environments. Furthermore, we assessed the influence of oxygen gradients on the balance of the recycling process and de novo synthesis of GSH in RBCs and underline the regulation of the glucose metabolism pathway between HMP and EMP. In addition, we also studied the effects of oxidation and phosphorylation of Band 3 on glucose metabolism and antioxidant capacity in RBCs under an *in vitro* oxidation environment.

## 2. Materials and Methods

### 2.1. Reagents and Antibodies

All chemicals and reagents were purchased from Sigma-Aldrich (St. Louis, MO, USA). Antibodies to GCLc (ab240379), GCLm (ab126704), Band 3 (ab108414), GAPDH (ab9485), and phosphotyrosine (ab179530) were obtained from Abcam (Cambridge, MA). Electrophoresis equipment and related supplies were obtained from Bio-Rad (USA). The spectrophotometer was purchased from Thermo Fisher Scientific (USA).

### 2.2. Blood Sampling and Generation of RBC Suspensions

Fresh blood was collected by venipuncture from healthy human volunteers (*n* = 6), and RBCs from each donor were isolated and washed three times in isotonic Hepes buffer, then resuspended to a hematocrit of 50% in Krebs buffer containing 2 g/l glucose (pH 7.4) for further experiments. All subjects gave their informed consent for inclusion before they participated in the study. The study was conducted in accordance with the Declaration of Helsinki, and the protocol was approved by the Ethics Committee of Binzhou Medical University (permit number: 2019-0004).

For the preparation of RBC ghosts, washed packed RBCs were lysed with ice-cold 5 mM sodium phosphate buffer (pH 8.0) containing 1 mM ethylene glycol tetraacetic acid (EGTA), 1 mM sodium orthovanadate, and 1 mM phenylmethylsulfonyl fluoride (lysis buffer) and incubated at 4°C for 10 min. RBC ghosts were washed thrice by centrifugation (25,000 g for 20 min at 4°C) to obtain “white” ghosts.

### 2.3. RBC Treatments

For manipulation of Hb conformation, washed RBCs (20-25 ml) were injected into a rotating glass tonometer (in a water bath, at 37°C), allowing controlled RBC oxygenation or deoxygenation, as well as sample extraction without atmospheric exposure as described previously [29]. The tonometer chamber was joined via a vacuum-tight, motorized couple to a gas source and purge ports to permit simultaneous chamber rotation and flushing by gas blended from O_2_, N_2_, and CO_2_ tanks. Washed RBCs were exposed for 25 min to either oxygenating conditions (21% O_2_, 5–9% CO_2_, balanced N_2_), yielding >97% HbSO_2_, or to deoxygenating conditions (0% O_2_, 5–9% CO_2_, balanced N_2_), yielding <10% HbSO_2_.

### 2.4. *In Vitro* Model of Oxidative Stress

A hypoxanthine/xanthine oxidase (HX/XO) system was used to generate a steady, gradable production of superoxide as described previously [5]. In brief, RBCs were portioned into aliquots and incubated with 1.5 mM HX and escalating doses of XO (0, 0.2, 0.4, or 0.8 U/ml) in a shaking heater block (37°C) for 0, 10, 20, 40, or 60 min. Following HX/XO exposure, RBCs were washed three times with phosphate-buffered solution.

Washed RBCs were incubated with dehydroepiandrosterone (DHEA) (5 *μ*M, 15 min, 37°C), a selective inhibitor of glucose-6-phosphate dehydrogenase (G6PD), to inhibit glucose utilization through HMP [30]. Washed RBCs were incubated with KA (15 *μ*M, 15 min, 37°C), a selective inhibitor of glyceraldehyde-3-phosphate dehydrogenase (G3PD), to inhibit glucose utilization through EMP [31]. Washed RBCs were preexposed to 100% carbon monoxide (CO) (5 min, room temperature) to lock the Hb conformation in the R state.

### 2.5. Detection of Free Thiol

The free thiol concentration (SH-radicals) of the RBC membrane was quantified according to the method of Yamaguchi et al. [32]. SH-radical levels were estimated by measuring absorbance at 415 nm after incubation at 37°C in a water bath for 15 min, and the contents of the SH-radical were determined using GSH as a standard.

### 2.6. Preparation and Examination of Erythrocytes by Scanning Electron Microscopy

The RBCs were fixed for 24 h in a 2% solution of glutaraldehyde. After that, the preparations were washed in phosphate buffer for 30 min, and then the material was dehydrated in a rising series of ethyl alcohol concentrations (50%, 70%, 80%, 95%, and 100%). Each sample was washed for 15 min in an appropriate concentration, with the material remaining in pure ethyl alcohol for 30 min. Next, the RBCs were dried for 12 h at room temperature and coated with gold using a sputter coater (Q150RS, Quorum Technologies, Laughton, UK). The material's ultrastructure was analyzed using a scanning microscope (Zeiss EVO LS15) with an SE1 detector, under high vacuum and accelerating voltage (acceleration voltage (EHT) = 10 kV). Individual forms of RBCs were ascribed morphological indices according to the Bessis scale [33].

### 2.7. Detection of ROS

The intracellular production of ROS was assessed by 2′,7′-dichlorofluorescein diacetate (H2DCF-DA) (Sigma-Aldrich, St Louis, MO, USA). The RBCs were collected and washed with PBS solution, H2DCF-DA was added (10 *μ*M), and the cells were incubated at 37°C for 30 min. After incubation, the cells were washed and analyzed by FCM.

### 2.8. Detection of Thiobarbituric Acid-Reactive Substances (TBARS)

The extent of lipid peroxidation of RBC membranes was estimated by measuring TBARS levels according to the method of Stocks and Dormandy [34]. TBARS levels were estimated by measuring absorbance at 532 nm after a reaction with thiobarbituric acid. Trichloroacetic acid extracts of RBC samples were used to circumvent the interference of proteins with TBARS determinations. Results were expressed as nanomoles per gram Hb.

### 2.9. Detection of Methemoglobin (MetHb)

Blood was centrifuged at 2,000 rpm at 4°C to remove plasma, passed through cotton to eliminate white cells, and washed three times with choline wash solution (180 mM choline, 1 mM MgCl_2_, 10 mM Tris-MOPS, pH 7.4 at 4°C, 320–340 mOsm). Red cell MetHb levels were determined as described by Kohn et al. [35].

### 2.10. Measurements of ATP

ATP concentrations were determined as we reported previously [36]. RBC samples were added to 10% trichloroacetic acid, vortexed, and placed on ice. The supernatants were combined with the substrates (glucose and NAD^+^) and enzymes (hexokinase and glucose-6-phosphate dehydrogenase) required for the enzymatic reaction to occur. The amount of NADH produced, which is proportional to the amount of ATP within the sample, was measured spectrophotometrically.

### 2.11. Measurements of NADPH

Total NADP(H) and NADPH were measured using a colorimetric assay kit (65349, Abcam). Hemoglobin and other proteins with MW greater than 30 kDa were removed from RBC lysates using centrifugal filters with a 30 kDa threshold, and filtrates were heated to 60°C for 30 minutes per the manufacturer's protocol to decompose NADP^+^ and permit measurement of the remaining NADPH. A detection reagent was added, and NADP(H) was monitored by absorbance at 450 nm for a period of 3 hours and quantified using standards [37].

### 2.12. Detection of FRAP

The ferric-reducing ability of plasma (FRAP) values were determined following the method of Benzie and Strain [38]. In brief, a total of 3 ml of the FRAP reagent was mixed with 100 *μ*l of plasma. The absorbance was read at 593 nm at an interval of 30 sec for 4 min. An aqueous solution of a known Fe^2+^ concentration in the range of 100-1,000 *μ*mol/l was used for calibration. Using the regression equation, the FRAP value (*μ*mol Fe(II) per liter) of the plasma was calculated.

### 2.13. Measurements of Enzyme Activity

RBCs were lysed with ice-cold 5 mM sodium phosphate buffer (pH 8.0) and vortexed. The RBC membrane was removed by centrifuging at 20,000 g for 20 min at 4°C. The supernatant was saved and used to measure cytosolic G6PD activity by a G6PD assay kit (#12581, CST, Massachusetts, USA) [39], and cytosolic GAPDH activity was measured by a GAPDH assay kit (AM1639, Life Technologies) [40]. The activities of CAT were assessed using the methods of Aebi [41]. The activities of SOD were assessed using the methods of Misra and Fridovich [42]. The activity of glutathione peroxidase (GPX) was measured using Paglia and Valentines's method [43]. The activity of glutathione reductase (GR) was assayed at 37°C using the method proposed by Flohe and Gunzler [44]. The activity of glutamate-cysteine ligase (GCL) was determined by the fluorescence assay described by Chen et al. [45].

### 2.14. Detection of GSH

The values of GSH, total free glutathione (TFG), and GSH/GSSG ratio were determined using a method described previously [36]. In brief, the amount (mole) of GSH and GSSG per RBC was determined via the standard curve specific to each run. The median of the three independently processed samples was taken to reflect the intracellular GSH and GSSG concentration of an individual. Erythrocyte TFG level was calculated as GSH + 2 × GSSG.

### 2.15. Detection of L-Cysteine Influx in RBC

The procedure for measuring L-cysteine influx was essentially the same as described earlier [46]. A total of 0.25 ml of washed packed erythrocytes was suspended in 1 ml of PBS containing 8 mM glucose and 10 mM L-cysteine and incubated for 1 hour at 37°C in a water bath. Influx rate was calculated by subtracting the control (erythrocytes incubated in PBS-glucose without L-cysteine for 1 hour at 37°C) free -SH concentration from free -SH concentration obtained following treatment with L-cysteine.

### 2.16. Electrophoresis and Immunoblotting Analyses of Plasma Membranes

Sodium dodecyl sulfate-polyacrylamide gel electrophoresis (SDS-PAGE) was conducted by heating the samples for 8 min at 100°C and loading 10 *μ*g of membrane proteins on a 5-15% linear acrylamide gradient gel (10 *μ*g protein/lane) according to Laemmli for protein staining by colloidal Coomassie Blue.

Western blot analyses were performed as previously described [47]. In short, total proteins were electrotransferred to PVDF membranes after having been separated by SDS-PAGE. Then, membranes were incubated with the indicated primary antibody overnight at 4°C after having been blocked in 5% BSA dissolved in TBST for 2 h at room temperature, followed by incubation with the appropriate HRP-linked secondary antibody 2 h at room temperature. ECL plus detection reagents (Beyotime, Shanghai, China) were used for visualized protein bands. The ImageJ gel analysis software was used for densitometric analysis.

### 2.17. Immunofluorescence and Image Analyses

RBCs were fixed with 4% paraformaldehyde and 0.05% glutaraldehyde in PBS and permeabilized in the same solution containing 0.05% Triton X-100. After being blocked with 3% BSA and 0.1% Tween 20 in PBS to block nonspecific protein binding, cells were treated with primary antibodies diluted in 10 mg/ml PBS for 1 h at room temperature. The sources of all antibodies were as mentioned above. Cells were washed at 3 min intervals thrice with gentle shaking, and then incubated for 1 h with secondary antibodies at 1 : 700 dilution in PBS, and washed thrice in PBS. Fluorescence was imaged using an Olympus IX71 microscope with a 63/1.25 oil immersion objective and equipped with a CCD camera (Olympus, Tokyo, Japan).

### 2.18. Measurement of Glycolysis Analysis

The extracellular acidification rate (ECAR) was measured in 500,000 purified RBCs per well using the Seahorse XF96 Extracellular Flux Analyzer (Seahorse Bioscience, North Billerica, MA, USA) as previous described [48]. After hypoxia and normoxia treatment of RBCs, ECAR measurements were made after sequential injection of glucose (10 mM) and 2-deoxyglucose (2-DG) (100 mM). Glycolysis capacity is the ECAR value after adding glucose minus the ECAR value after adding 2-DG.

### 2.19. Statistical Analysis

The experimental data were expressed as mean ± SEM. The statistical analysis was performed by one-way ANOVA followed by Tukey's test for multiple comparisons using the SPSS software (SPSS 19.0; IBM Corporation, Armonk, NY, USA). A value of *P* < 0.05 was considered statistically significant.

## 3. Results

### 3.1. Hypoxia Promotes the Loss of Membrane Free Thiol in RBCs Exposed to Oxidative Stress

**Figure 1:**
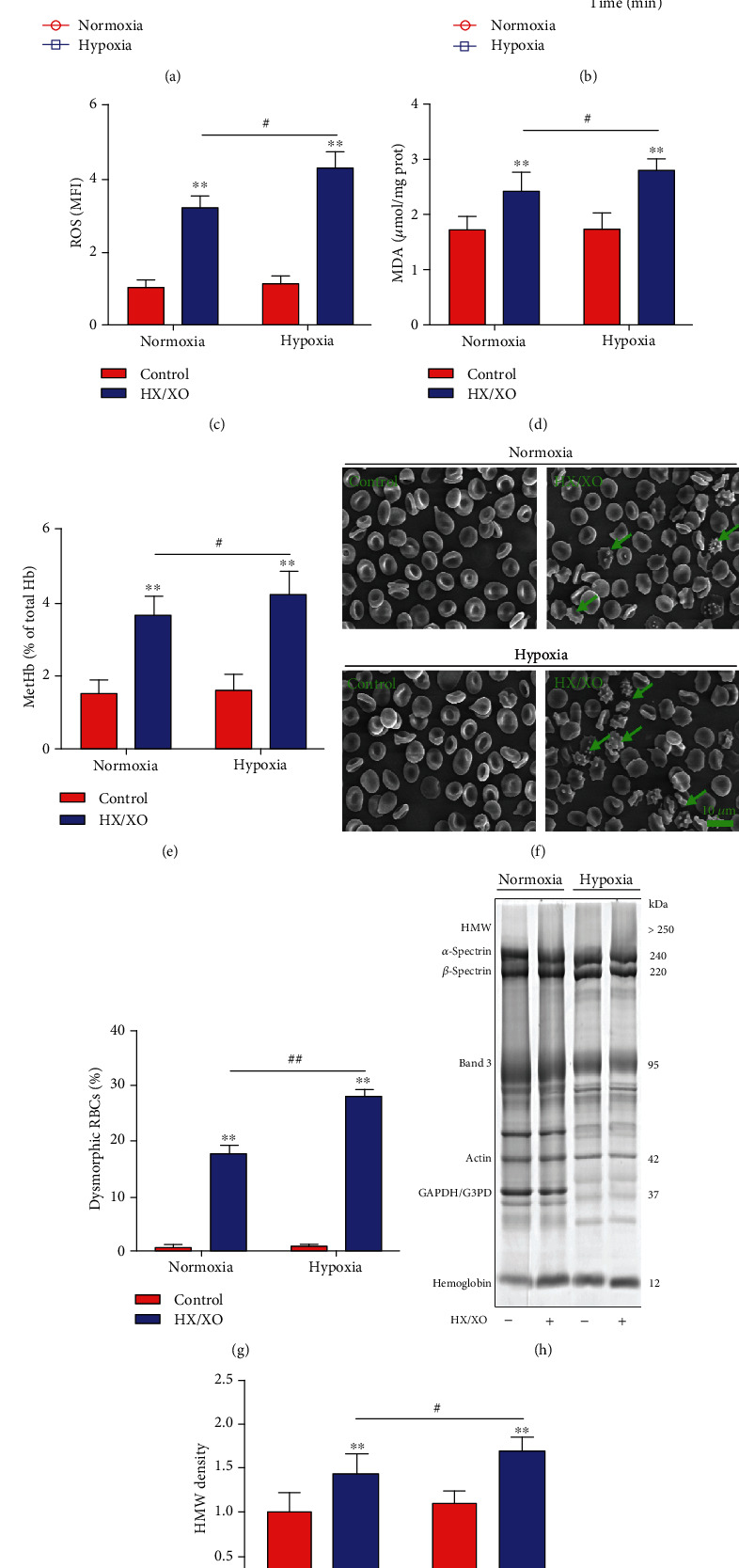
Loss of RBC membrane free thiol and increased oxidative stress injury in RBC preparations exposed to oxidative stress. (a) Levels of membrane free thiol in normoxic and hypoxic RBCs following gradient concentration HX/XO treatment (45 min exposure). (b) Levels of membrane free thiol in normoxic and hypoxic RBCs following gradient time HX/XO treatment (1.5 mM HX; 0.4 U/ml XO). (c–e) Levels of ROS, MDA, and MetHb in normoxic and hypoxic RBCs after HX/XO exposure (1.5 mM HX; 0.4 U/ml XO; 30 min). (f and g) The representative normoxic and hypoxic RBC photos were obtained using a scanning electron microscope; the percentages of dysmorphic RBCs are shown. Green arrows indicate dysmorphic RBCs. Scale bars = 10 *μ*m. (h and i) SDS-PAGE analyses of erythrocyte membrane proteins; the densitometric analyses of the HMW band (>250 kDa) are shown. Data represent the mean scores ± SEM of at least three independent experiments. ^∗^*P* < 0.05 and ^∗∗^*P* < 0.01 for normoxic or hypoxic RBCs *vs.* corresponding RBCs with HX/XO exposure. ^#^*P* < 0.05 for normoxic RBCs *vs.* corresponding hypoxic RBCs.

In a series of *in vitro* experiments, we tested the relationship among oxygen gradients and free thiol radicals in RBC membranes following graded oxidative stress (45 min exposure). As shown in Figure 1(a), the loss of free thiol in both hypoxic and normoxic RBCs progressed with the severity of oxidative insult. Moreover, with increasing exposure time in a stable oxidative stress condition (HX, 1.5 mM; XO, 0.4 U/ml), the loss of free thiol radicals was also significantly greater in hypoxic RBCs than in normoxic RBCs (Figure 1(b)).

### 3.2. Hypoxia Promotes Oxidative Stress Injury of RBCs

Under the same condition of *in vitro* oxidation (HX, 1.5 mM; XO, 0.4 U/ml; 30 min), we detected the degree of oxidative damage of RBCs under hypoxia or normoxia conditions. As shown in Figures 1(c)–1(e), in the presence of HX/XO, the levels of ROS, MDA, and MetHb increased markedly in RBCs of both the hypoxia and normoxia groups. Interestingly, the hypoxia group showed more significantly higher levels of ROS, MDA, and MetHb compared with the normoxia group.

Scanning electron microscopy showed that the flattened biconcave disc morphology of RBCs was damaged and dysmorphic RBCs were present (e.g., surface blebbing typical of acanthocytes) after HX/XO exposure (Figure 1(f)). In addition, there was a considerably higher percentage of dysmorphic RBCs in the hypoxia group than in the normoxia group after exposure to HX/XO (Figure 1(g)). SDS-PAGE analyses of RBC membrane proteins show that after exposure to HX/XO, the gray value of the high-molecular-weight (HMW) band (>250 kDa) increased, especially in the hypoxia group (Figures 1(h) and 1(i)).

### 3.3. Hypoxia Limits the Antioxidant Capacity of RBCs Exposed to Oxidative Stress

**Figure 2:**
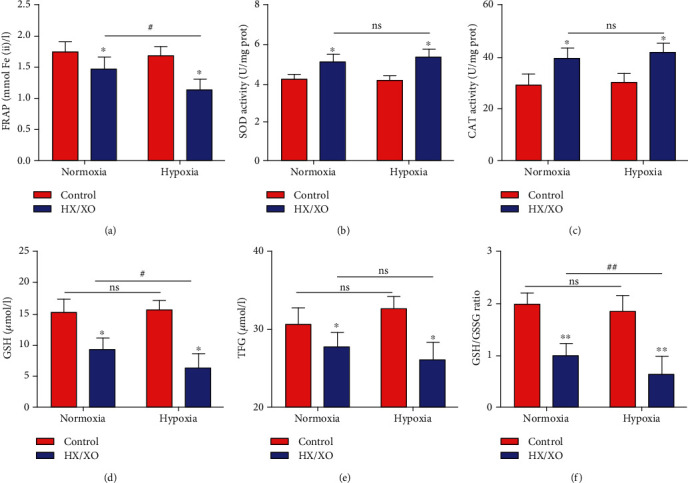
Hypoxia limits the antioxidant capacity of RBCs exposed to oxidative stress. Levels of FRAP (a), SOD (b), and CAT (c) in normoxic and hypoxic RBCs after HX/XO exposure (1.5 mM HX; 0.4 U/ml XO; 30 min). Glutathione-related parameters, including GSH (d), TFG (e), and GSH/GSSG ratio (f) in normoxic and hypoxic RBCs. Data represent the mean scores ± SEM of at least three independent experiments. ^∗^*P* < 0.05 and ^∗∗^*P* < 0.01 for normoxic or hypoxic RBCs *vs.* corresponding RBCs with HX/XO exposure. ^#^*P* < 0.05 for normoxic RBCs *vs.* corresponding hypoxic RBCs.

In order to determine the reason for the increased oxidative damage of RBCs in the hypoxia group, we analyzed the antioxidant capacity of RBCs in the hypoxia and normoxia groups under *in vitro* oxidation (HX, 1.5 mM; XO, 0.4 U/ml; 30 min). As shown in Figure 2(a), the FRAP value significantly decreased in both hypoxic and normoxic RBCs after HX/XO exposure. And the hypoxia group showed significantly lower levels of FRAP compared with the normoxia group.

To determine the reason for the change of the antioxidant capacity of RBCs in different oxygen gradients, we analyzed the activities of the main enzymatic and nonenzymatic antioxidants in RBCs. As shown in Figures 2(b) and 2(c), the activities of SOD and CAT significantly increased in both hypoxic and normoxic RBCs after HX/XO exposure. And there were no significant differences between the hypoxia and normoxia groups.

In the aspect of nonenzymatic antioxidants, we found that HX/XO exposure induced significantly decreased GSH and TFG levels and GSH/GSSG ratio in both hypoxic and normoxic RBCs (Figures 2(d)–2(f)). In addition, the GSH level and GSH/GSSG ratio in the hypoxia group was significantly decreased compared with those in the normoxia group after HX/XO exposure. Under the condition of no oxidation, the TFG level of RBCs in the hypoxia group was slightly higher (no significant) than that in the normoxia group, but there were no significant differences between the two groups after HX/XO exposure.

### 3.4. Hypoxia Downregulates the GSH Synthesis of RBCs Exposed to Oxidative Stress

**Figure 3:**
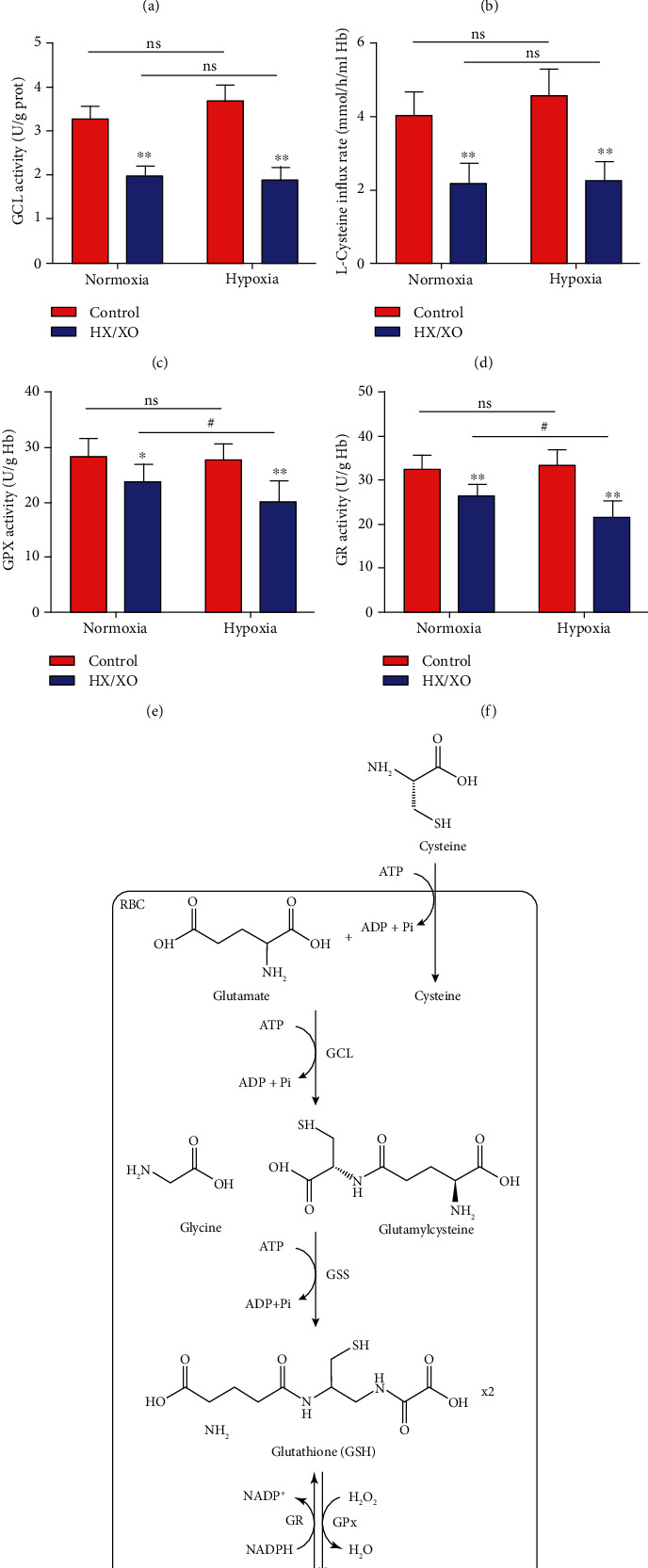
Hypoxia downregulates the GSH synthesis of RBCs exposed to oxidative stress. The expressions of GCLc (a) and GCLm (b) in normoxic and hypoxic RBCs after HX/XO exposure (1.5 mM HX; 0.4 U/ml XO; 30 min). (c) The activities of GCL in normoxic and hypoxic RBCs. (d) The L-cysteine influx rate in normoxic and hypoxic RBCs. The activities of GPX (e) and GR (f) in normoxic and hypoxic RBCs. (g) Schematic of GSH and GSSG synthesis. Data represent the mean scores ± SEM of at least three independent experiments. ^∗^*P* < 0.05 and ^∗∗^*P* < 0.01 for normoxic or hypoxic RBCs *vs.* corresponding RBCs with HX/XO exposure. ^#^*P* < 0.05 for normoxic RBCs *vs.* corresponding hypoxic RBCs.

We further explored the changes of GSH synthesis-related enzyme activities in RBCs under different oxygen gradients. As shown in Figures 3(a)–3(c), HX/XO exposure (HX, 1.5 mM; XO, 0.4 U/ml; 30 min) induced significantly decreased GCLc and GCLm expression and GCL activity in both the hypoxia and normoxia groups. In addition, as the main factor regulating the synthesis rate of glutathione, the L-cysteine transport was also decreased significantly after HX/XO exposure (Figure 3(d)). Remarkably, the GCL activity and L-cysteine transport of RBCs in the hypoxia group were slightly higher (not significant) than those in the normoxia group under HX/XO unexposed conditions. However, there were no significant differences between the hypoxia and normoxia groups after HX/XO exposure.

Similarly, we found that the activities of GPX and GR decreased significantly in both the hypoxia and normoxia groups after HX/XO exposure (Figures 3(e) and 3(f)). By contrast, after HX/XO exposure, the activities of GPX and GR were significantly lower in the hypoxia group compared with the normoxia group.

### 3.5. Hypoxia Downregulates the Glucose Metabolism of RBCs Exposed to Oxidative Stress

**Figure 4:**
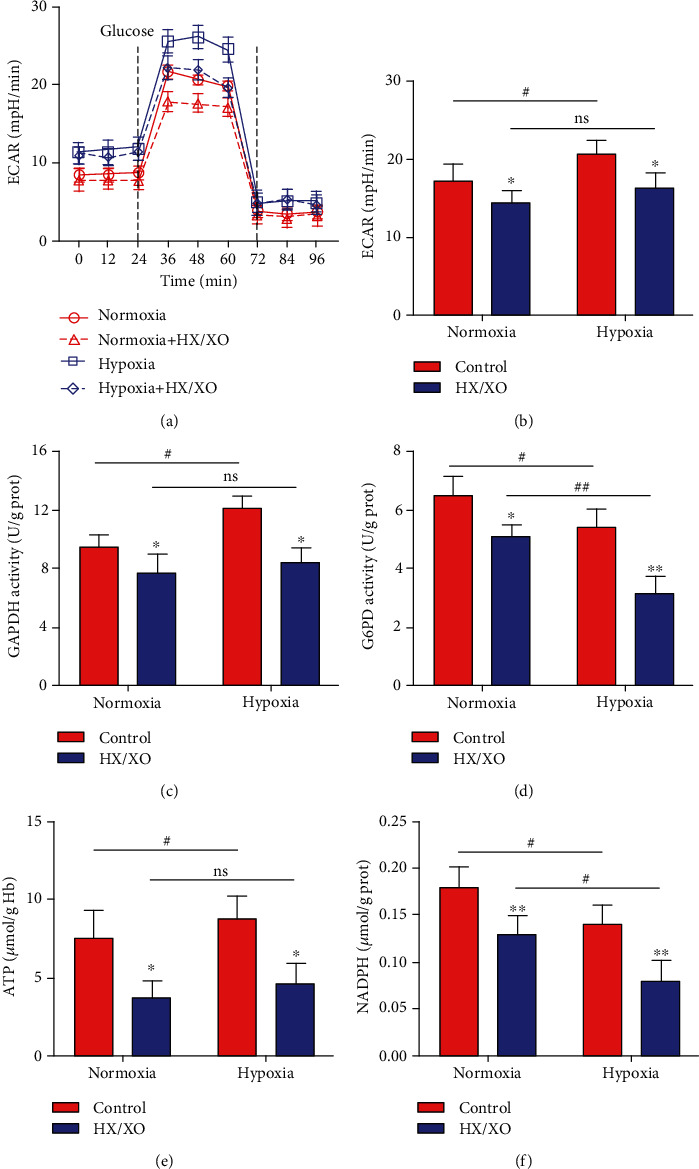
Hypoxia downregulates the glucose metabolism of RBCs exposed to oxidative stress. (a and b) ECAR in normoxic and hypoxic RBCs after HX/XO exposure (1.5 mM HX; 0.4 U/ml XO; 30 min). (c) The activity of GAPDH, the rate-limiting enzyme of EMP, in normoxic and hypoxic RBCs. (d) The activity of G6PD, the rate-limiting enzyme of HMP, in normoxic and hypoxic RBCs. The levels of ATP (e) and NADPH (f) in normoxic and hypoxic RBCs. Data represent the mean scores ± SEM of at least three independent experiments. ^∗^*P* < 0.05 and ^∗∗^*P* < 0.01 for normoxic or hypoxic RBCs *vs.* corresponding RBCs with HX/XO exposure. ^#^*P* < 0.05 for normoxic RBCs *vs.* corresponding hypoxic RBCs.

ATP and NADPH provided by the EMP and HMP pathways of RBCs are required for the de novo synthesis and recycling regeneration of GSH (Figure 3(g)). Therefore, we analyzed the effects of hypoxia on the glucose metabolism of RBCs under oxidative stress. As shown in Figures 4(a) and 4(b), the glycolysis ability of RBCs significantly decreased in both the hypoxia and normoxia groups after HX/XO exposure. Interestingly, the glycolysis ability of RBCs in the hypoxia group was significantly higher than that in the normoxia group under HX/XO unexposed conditions, but there were no significant differences between the two groups after HX/XO exposure. Similarly, we found the same trend of changes in the activity of GAPDH, the rate-limiting enzyme of EMP (Figure 4(c)).

On the other hand, the activity of G6PD, the rate-limiting enzyme of HMP, also decreased significantly in both the hypoxia and normoxia groups after HX/XO exposure (Figure 4(d)). However, compared with the normoxia group, the significantly lower glycolysis ability in the hypoxia group became more significant after HX/XO exposure.

As a result, we detected significantly reduced levels of ATP and NADPH in RBCs of both the hypoxia and normoxia groups after HX/XO exposure (Figures 4(e) and 4(f)). Compared with the normoxia group, there was no significant difference in the ATP level of RBCs in the hypoxia group after HX/XO exposure. In contrast, there was a significantly decreased NADPH level in the hypoxia group compared with the normoxia group after HX/XO exposure. And there was a significantly increased ATP level in the hypoxia group compared with the normoxia group under HX/XO unexposed conditions.

### 3.6. Band 3 Oxidative Phosphorylation Downregulates the Synthesis of GSH in Hypoxic RBCs Exposed to Oxidative Stress

**Figure 5:**
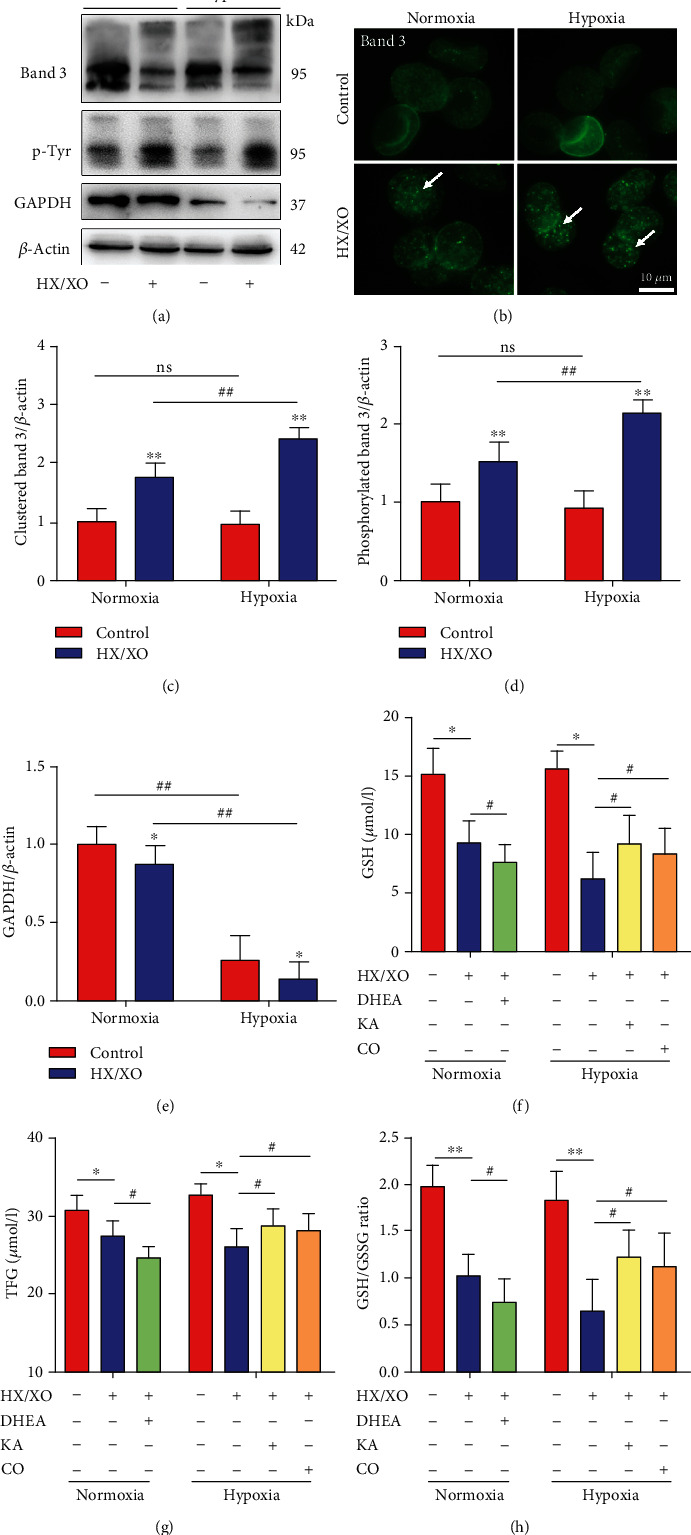
Hypoxia promotes oxidative phosphorylation of Band 3 in RBCs exposed to oxidative stress. (a) The expressions of G3PD, Band 3, and phosphotyrosine in normoxic and hypoxic RBCs were evaluated by Western blot. (b) Fluorescent micrographs for normoxic and hypoxic RBCs after immunostaining with monoclonal antibodies to Band 3. The arrows indicate protein clusters after HX/XO exposure. Bars = 10 *μ*m. (c) Densitometric analyses of immunoblots of Band 3. (d) Densitometric analyses of immunoblots of phosphotyrosine protein. (e) Densitometric analyses of immunoblots of GAPDH. Glutathione-related parameters, including GSH (f), TFG (g), and GSH/GSSG ratio (h) in normoxic and hypoxic RBCs with pretreatment of DHEA (inhibitor of HMP key enzyme G6PD), KA (inhibitor of EMP key enzyme GAPDH), or CO (stabilizing Hb conformation). Data represent the mean scores ± SEM of at least three independent experiments. ^∗^*P* < 0.05 and ^∗∗^*P* < 0.01 for normoxic or hypoxic RBCs *vs.* corresponding RBCs with HX/XO exposure. ^#^*P* < 0.05 for normoxic RBCs *vs.* corresponding hypoxic RBCs.

The generation of NADPH (HMP) or ATP, NADH, and 2,3-DPG (EMP) shifts with the RBC oxygen content because of the competition between key EMP enzymes and deoxy-Hb for binding to the cytoplasmic domain of the Band 3 membrane protein. To examine whether Band 3 is involved in the abnormal GSH synthesis induced by hypoxia, we detected the oxidative crosslinking and tyrosine phosphorylation (p-Tyr) of Band 3 in hypoxic and normoxic RBCs after HX/XO exposure (HX, 1.5 mM; XO, 0.4 U/ml; 30 min). Band 3 immunoblotting is shown in Figure 5(a). Densitometric analyses showed significantly elevated clustering products (clustered Band 3) and p-Tyr in Band 3 in both the hypoxia and normoxia groups after HX/XO exposure (Figures 5(c) and 5(d)). In addition, the levels of clustered and p-Tyr Band 3 were significantly higher in the hypoxia group compared with the normoxia group after HX/XO exposure. Subsequent microscopic analysis of Band 3 verified the presence of Band 3 aggregates in RBCs formed after HX/XO exposure (Figure 5(b)). Furthermore, we found that the level of membrane-bound GAPDH decreased significantly with the decrease of oxygen content, and oxidative stress further inhibited the binding of GAPDH to the membrane in both hypoxic and normoxic RBCs (Figure 5(e)). These results suggest that oxidative stress-induced oxidative phosphorylation of Band 3 may interfere with the competitive binding of deoxy-Hb and GAPDH with Band 3, and further lead to the abnormal glucose metabolism and GSH synthesis of RBCs.

To verify the hypothesis, we locked the Hb conformation in the R state with 100% carbon monoxide (CO) to block the release of the EMP enzyme from Band 3 in hypoxic RBCs. As shown in Figures 5(f)–5(h), compared with the untreated group, the levels of GSH and TFG and GSH/GSSG ratio significantly increased in CO-pretreated hypoxic RBCs with HX/XO exposure. And similar results were also found in hypoxic RBCs pretreated with the GAPDH inhibitor KA to block glucose metabolism via EMP. On the contrary, blocking glucose metabolism via HMP with the G6PD inhibitor DHEA significantly decreased the levels of GSH and TFG and GSH/GSSG ratio in normoxic RBCs with HX/XO exposure.

## 4. Discussion

The research we have done suggests a decreased antioxidant capacity and an increased risk of oxidative damage in hypoxic RBCs. Under *in vitro* oxidation conditions, we found the dysfunction of GSH synthesis in both de novo synthesis and the recycling process in hypoxic RBCs. In addition, we provide evidence that the main reason for the significantly decreased GSH content and antioxidant capacity of hypoxic RBCs compared with the normoxia group is the disturbance of the recycling process but not the de novo synthesis of GSH. Further investigation reflects an underlying molecular mechanism by which oxidative phosphorylation of Band 3 blocked the HMP pathway and decreased NADPH production aggravating the dysfunction of GSH synthesis in hypoxic RBCs under oxidative conditions.

RBCs are permanently in danger of oxidative stress across their lifetime due to the high cellular concentration of oxygen and Hb [49, 50]. It also leads to a series of changes, such as crosslinking of membrane protein, reduction of deformability, and release of MetHb, which further aggravates the free radical burst and causing secondary damage to the body [51–53]. In this study, we also found that oxidative stress induced membrane protein crosslinking and the presence of dysmorphic RBCs under *in vitro* oxidation conditions. In addition, the hypoxia group showed more serious oxidative stress injury compared with the normoxia group. This is in line with the previous research report that significantly increased oxidative stress of RBCs in the human body under a high-altitude hypoxic environment [54].

It is well known that RBCs possess enzymatic and nonenzymatic antioxidants, mainly composed of CAT, SOD, and GSH, to remove free radicals produced inside and outside cells [55, 56]. In this study, we found a significantly increased SOD and CAT activity in RBCs under *in vitro* oxidation conditions. But on the contrary, the antioxidant capacity of RBCs was significantly decreased accompanied by the increase of MDA and MetHb levels in RBCs under *in vitro* oxidation conditions. This may be due to the continuous production of oxidation products after the antioxidant capacity reaches the upper limit, which in turn destroys the antioxidant metabolism of RBCs. Interestingly, we also found that the antioxidant capacity of RBCs in the hypoxia group was significantly lower than that in the normoxia group. And this result was in agreement with previous research that hypoxia limits the antioxidant capacity in RBCs [5]. As there were no significant differences in both SOD and CAT activities between the hypoxia and normoxia groups, we speculate that the decrease of GSH activity was the key reason for the further decreased antioxidant capacity in hypoxic RBCs under *in vitro* oxidation conditions. In the present study, we found significantly decreased GSH and TFG levels and GSH/GSSG ratio in RBCs of both the hypoxia and normoxia groups under *in vitro* oxidation conditions, and the decrease of the GSH level and GSH/GSSG ratio was more significant in the hypoxia group. On the contrary, there was no significant difference in TFG level between the hypoxia and normoxia groups after HX/XO exposure. These results indicated that although both the recycling process and de novo synthesis of GSH in RBCs were damaged by oxidative stress, the recycling process is more susceptible to dysfunction in a hypoxic environment.

RBC GSH content is largely determined by intracellular production through de novo synthesis by glutamic acid, cysteine, and glycine, a process catalyzed by GCL and glutathione synthetase (GSS), as well as through the recycling regeneration between GSH and GSSG, a process catalyzed by GPX and GR (Figure 3(g)). In addition, previous studies also found that despite the fact that three amino acids were required for GSH synthesis, L-cysteine availability was the rate-limiting step of GSH synthesis [36, 57]. In the present study, we found no significant difference in GCLc and GCLm expression, GCL activity, and L-cysteine transport between normoxic and hypoxic RBCs after HX/XO exposure. However, the activities of GPX and GR are significantly lowered in hypoxic RBCs compared with the normoxia group under *in vitro* oxidation conditions. These findings verify our previous speculation that compared with the de novo synthesis, the recycling process of GSH is more vulnerable to oxidative stress injury in a hypoxic environment.

Previous studies have shown that both the L-cysteine transport and the de novo synthesis of GSH catalyzed by GCL in RBCs requires the EMP pathway to provide ATP [58, 59]. On the other hand, the recycling process between GSH and GSSG in RBCs requires the HMP pathway to provide NADPH [5]. Therefore, the change of glucose metabolism between EMP and HMP potentially regulate the balance of de novo synthesis and the recycling process of GSH in RBCs. In the present study, we found a significantly higher glycolysis ability in hypoxic RBCs under the condition of no oxidation. However, there were no significant differences between hypoxic and normoxic RBCs after HX/XO exposure. As a result, there were no significant differences in the ATP level between hypoxic and normoxic RBCs under oxidative conditions. On the contrary, the activity of the HMP key enzyme and the level of NADPH in the hypoxic RBCs were significantly lower than that in the normoxia group under both oxidation and nonoxidation conditions. In addition, under *in vitro* oxidation conditions, we found significantly increased levels of GSH and TFG and GSH/GSSG ratio in hypoxic RBCs pretreated with the GAPDH inhibitor KA to block glucose metabolism via EMP. On the contrary, blocking glucose metabolism via HMP with the G6PD inhibitor DHEA significantly decreased the levels of GSH and TFG and GSH/GSSG ratio in normoxic RBCs with HX/XO exposure. Our results in this study confirmed these previous findings that glucose metabolism in RBCs is characterized by O_2_-responsive variations in flux through EMP or HMP [4]. And the differences of the anti-interference ability to oxidative stress between the EMP and HMP pathways in RBCs under different oxygen gradients potentially regulate the pathway of GSH synthesis and lead to the differences in antioxidant capacity.

**Figure 6:**
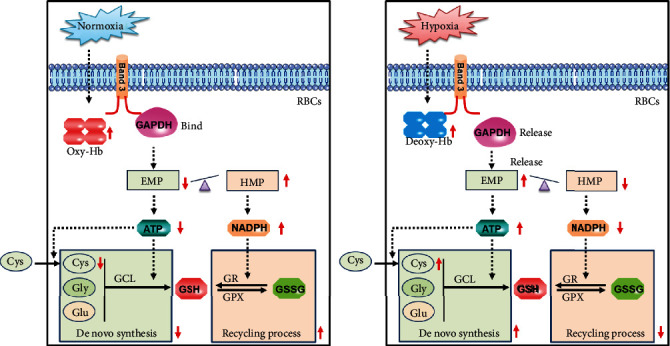
Schematic illustration of O_2_ gradient-mediated de novo synthesis and recycling process of GSH in RBCs. Compared with a normoxic environment, the concentration of deoxy-Hb in RBCs increased in a hypoxic environment, and binds to the cytoplasmic domain of Band 3. This promotes the release of GAPDH from Band 3 and shifts glucose metabolism from HMP to EMP, and in turn leads to the generation of more ATP and less NADPH. Finally, hypoxia limited the antioxidant capacity of RBCs by downregulating the cycle regeneration of GSH.

Deoxygenated RBCs are known to undergo reversible phosphorylation of Band 3 and improve the binding with increased deoxy-Hb, which inhibit the binding of GAPDH with Band 3 and improve the glucose metabolism flux through EMP [60–62]. When RBCs are exposed to an environment with high oxygen content, the decrease of deoxy-Hb is accompanied with Band 3 dephosphorylation; this improves the binding of GAPDH with Band 3 and improves the glucose metabolism flux through HMP and provide more NADPH for the GSH recycling process (Figure 6). In this study, we verify the above regulatory mechanism by blocking the release of EMP enzymes from Band 3 with CO treatment, and found improved GSH synthesis in hypoxic RBCs. However, under a severe oxidative stress environment, irreversible oxidative phosphorylation of Band 3 will lead to the difficulty in binding and releasing of both deoxy-Hb and/or GAPDH with Band 3, which results in the weakening of the above regulatory functions. Here, we also detected a significant increase in the levels of oxidation and p-Tyr of Band 3 accompanied with a significant decrease of membrane-binding GAPDH in hypoxic RBCs. This is consistent with our result that shows an increased glycolysis ability and decreased NADPH level in hypoxic RBCs with HX/XO exposure. Our research suggests that we should pay more attention to avoid serious oxidative stress in a hypoxic environment such as high-altitude hypoxia or exhaustive exercise.

## 5. Conclusion

In summary, we presented evidence that the antioxidant capacity of RBCs is reduced and more vulnerable to oxidative damage under hypoxia conditions. The disturbance of the recycling process, but not de novo synthesis of GSH, accounted for the decreased antioxidant capacity in hypoxic RBCs. In addition, the transition from the HMP pathway to the EMP pathway of glucose metabolism, which was interfered with by the oxidative phosphorylation of Band 3, is also involved in the regulation of the antioxidant capacity of RBCs under different oxygen gradients.

## Data Availability

The data that support the findings of this study are available from the corresponding author upon request.

## Abbreviations

RBCs: Red blood cells
HX/XO: Hypoxanthine/xanthine oxidase
ROS: Reactive oxygen species
GSH: Glutathione
TFG: Total free glutathione
EMP: Embden-Meyerhof pathway
HMP: Hexose monophosphate pathway
CAT: Catalase
SOD: Superoxide dismutase
FRAP: Ferric-reducing ability of plasma
GPX: Glutathione peroxidase
GR: Glutathione reductase
GCL: Glutamate-cysteine ligase
ECAR: Extracellular acidification rate.
