# Correction to “Downregulated Recycling Process but Not De Novo Synthesis of Glutathione Limits Antioxidant Capacity of Erythrocytes in Hypoxia”

**DOI:** 10.1155/omcl/9879523

**Published:** 2026-04-17

Y. Wang, N. Zhao, Y. Xiong, J. Zhang, D. Zhao, Y. Yin, L. Song, Y. Yin, J. Wang, X. Luan, and Y. Xiong, “Downregulated Recycling Process but Not De Novo Synthesis of Glutathione Limits Antioxidant Capacity of Erythrocytes in Hypoxia,” *Oxidative Medicine and Cellular Longevity*, 2020, 7834252, https://doi.org/10.1155/2020/7834252.

In the article, there is an error in Figure 5, in which the ß‐actin bands in Figure 5a are identical to the AKT bands shown in Figure 3b of the author’s other work [1]. The correct Figure 5 is shown below:

**Figure 5:**
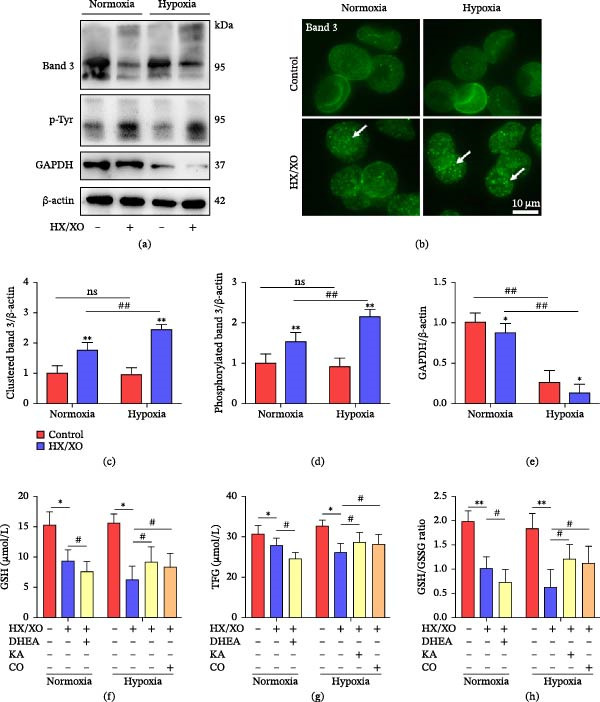
Hypoxia promotes oxidative phosphorylation of Band 3 in RBCs exposed to oxidative stress. (a) The expressions of G3PD, Band 3, and phosphotyrosine in normoxic and hypoxic RBCs were evaluated by Western blot. (b) Fluorescent micrographs for normoxic and hypoxic RBCs after immunostaining with monoclonal antibodies to Band 3. The arrows indicate protein clusters after HX/XO exposure. Bars = 10 μm. (c) Densitometric analyses of immunoblots of Band 3. (d) Densitometric analyses of immunoblots of phosphotyrosine protein. (e) Densitometric analyses of immunoblots of GAPDH. Glutathione‐related parameters, including GSH (f), TFG (g), and GSH/GSSG ratio (h) in normoxic and hypoxic RBCs with pretreatment of DHEA (inhibitor of HMP key enzyme G6PD), KA (inhibitor of EMP key enzyme GAPDH), or CO (stabilizing Hb conformation). Data represent the mean scores ± SEM of at least three independent experiments.  ^∗^ *P*  < 0.05 and  ^∗∗^ *P*  < 0.01 for normoxic or hypoxic RBCs vs. corresponding RBCs with HX/XO exposure. ^#^ *P*  < 0.05 for normoxic RBCs vs. corresponding hypoxic RBCs.

Additionally, the authors have requested to add the following clarification to the Figure 3 legend:

“Note: The target proteins (GCLc and GCLm) and the internal loading control (β‐actin) were analyzed using the same experimental replicates and protein lysates from a single experimental run; therefore, they share a common loading control panel to ensure consistent normalization.“

We apologize for these errors.
